# Development of a Novel Targeted Metabolomic LC-QqQ-MS Method in Allergic Inflammation

**DOI:** 10.3390/metabo12070592

**Published:** 2022-06-25

**Authors:** David Obeso, Nuria Contreras, Mariana Dolores-Hernández, Teresa Carrillo, Coral Barbas, María M. Escribese, Alma Villaseñor, Domingo Barber

**Affiliations:** 1 Department of Chemistry and Biochemistry, Facultad de Farmacia, Centre for Metabolomics and Bioanalysis (CEMBIO), Universidad San Pablo CEU, CEU Universities, Urbanización Montepríncipe, 28003 Madrid, Spain; nuria.contrerasgomez1@ceu.es (N.C.); may34@comunidad.unam.mx (M.D.-H.); cbarbas@ceu.es (C.B.); alma.villasenor@ceu.es (A.V.); 2 Department of Basic Medical Sciences, Facultad de Medicina, Institute of Applied Molecular Medicine (IMMA), Universidad San Pablo CEU, CEU Universities, Urbanización Montepríncipe, 28003 Madrid, Spain; mariamarta.escribesealonso@ceu.es (M.M.E.); domingo.barberhernandez@ceu.es (D.B.); 3 Laboratory of Pharmaceutical Development Tests, Multidisciplinary Research Unit (UIM), Faculty of Higher Education Cuautitlán, National Autonomous University of Mexico, Carr. Cuautitlán-Teoloyucan Km 2.5, San Sebastián Xhala, Cuautitlán Izcalli 54714, Mexico; 4 Servicio de Alergia, Hospital Universitario de Gran Canaria Doctor Negrín, 35010 Las Palmas de Gran Canaria, Spain; tcardia@gobiernodecanarias.org

**Keywords:** allergy, asthma, anaphylaxis, targeted metabolomics, liquid chromatography coupled to a triple quadrupole mass spectrometry, dynamic multiple reaction monitoring, allergic inflammation

## Abstract

The transition from mild to severe allergic phenotypes is still poorly understood and there is an urgent need of incorporating new therapies, accompanied by personalized diagnosis approaches. This work presents the development of a novel targeted metabolomic methodology for the analysis of 36 metabolites related to allergic inflammation, including mostly sphingolipids, lysophospholipids, amino acids, and those of energy metabolism previously identified in non-targeted studies. The methodology consisted of two complementary chromatography methods, HILIC and reversed-phase. These were developed using liquid chromatography, coupled to triple quadrupole mass spectrometry (LC-QqQ-MS) in dynamic multiple reaction monitoring (dMRM) acquisition mode and were validated using ICH guidelines. Serum samples from two clinical models of allergic asthma patients were used for method application, which were as follows: (1) corticosteroid-controlled (ICS, *n* = 6) versus uncontrolled (UC, *n* = 4) patients, and immunotherapy-controlled (IT, *n* = 23) versus biologicals-controlled (BIO, *n* = 12) patients. The results showed significant differences mainly in lysophospholipids using univariate analyses in both models. Multivariate analysis for model 1 was able to distinguish both groups, while for model 2, the results showed the correct classification of all BIO samples within their group. Thus, this methodology can be of great importance for further understanding the role of these metabolites in allergic diseases as potential biomarkers for disease severity and for predicting patient treatment response.

## 1. Introduction

Allergy disorders have been steadily increasing during the last few decades [1,2]. Along with this increase, an extensive arsenal of new therapeutic options has been developed. These options, on top of widely used symptomatic treatments, such as antihistamines or local corticoids and allergen-specific immunotherapy (AIT), are progressively incorporating a range of new biological drugs that target the main Th2 inflammatory routes. Thus, interleukin (IL)-4, IL-13, IL-5, thymic stromal lymphopoietin (TSLP) and immunoglobulin (Ig)E are targets of monoclonal antibody-based therapies, either directly or by blocking specific receptors [3,4]. However, the incorporation of new therapies has not been accompanied by personalized diagnostic approaches and the order in which the therapeutic options are implemented is subject to trial/error strategies or governed by subjective decisions. Moreover, the progression from mild to severe phenotypes is poorly understood and prevention and diagnosis strategies are urgently required. Over the last few years, different biomarkers have been postulated for allergic patient management [5], with some promising results, although these are still insufficient and better biomarkers are urgently needed [6]. For this reason, new omics-based strategies to discover novel biomarkers are being considered [7].

Defining allergic disease severity is difficult [8]. Severe and life-threatening anaphylactic reactions can happen with very limited systemic inflammation, while severe allergic asthmatic patients suffer from life-threatening exacerbations linked to perennial inflammation and progressive airway remodeling [9,10]. In addition, the combination of allergic phenotypes called endo-phenotypes and comorbidities makes the diagnosis of these patients difficult [11,12,13]. This may be further complicated by the fact that allergic patients are usually under medication that can also interfere with their diagnosis.

Metabolomics is the science that focuses on the metabolism of living organisms. It is a useful tool to discover new molecular mechanisms in diseases through the application of a non-targeted approach [8,14,15].

In recent years, by using non-targeted metabolomics in combination with transcriptomics and proteomics in different allergic disease severity models, we have identified new biological systems and associated promising potential biomarker candidates [16,17,18,19,20]. Furthermore, the combined analysis of these studies has allowed us to identify metabolites potentially related to energy metabolism, sphingolipid, phospholipid pathways, and platelet function associated with the severe phenotype [21,22]. We have also extensively studied these new routes in connection with allergic disease severity [23], as these are the main inflammatory routes shared by diverse severity models of chronic inflammation. Complementarily, in the anaphylactic model, new metabolites, such as cortisol and oleamide, have been identified as new promising disease classifiers [17].

These non-targeted studies are an important initial platform to elucidate their role in monitoring treatments in patients with different allergic phenotypes. However, targeted analysis of metabolites obtained from non-targeted methodologies is usually hampered by the availability of commercial standards, which are needed to develop new analytical methodologies [7,24,25]. Nonetheless, after an extensive review of the availability of standards and the pathways involved, this study aims to develop a targeted methodology to analyze up to 36 metabolites related to allergic inflammation. The methodology has been validated and applied to the following two patient models: (1) female patients with allergic asthma controlled with inhaled corticosteroids (ICS) and uncontrolled (UC), and (2) male and female patients with allergic asthma controlled with sublingual immunotherapy (IT) and biologics (BIO) controlled with omalizumab. This targeted methodology can be used to analyze new samples of allergic patients to further validate the findings obtained in the previous non-targeted analysis and could help elucidate the role of these metabolites in different allergic phenotypes.

## 2. Results

### 2.1. Optimization of the Parameters for HILIC and Reversed-Phase Methods in LC-QqQ-MS

Metabolites from three non-targeted metabolomics studies were selected (Table S1) [16,17,18]. These encompassed sphingolipids (sphinganine-1-phosphate (SPA-1P), sphinganine-C17, sphingosine and sphingosine-1-phosphate (S1P)), lysophospholipids (lysophosphocholine (LPC) 14:0, LPC 16:0, LPC 17:0, LPC 17:1, LPC 18:0, LPC 18:1, LPC 19:0, lysophosphoethanolamine (LPE) 18:0, lysophosphatidylinositol (LPI) 16:0 and LPI 20:4), amino acids (arginine, betaine, creatine, creatinine, leucine/isoleucine, phenylalanine and proline), fatty acids (arachidonic acid, lauric acid, oleic acid and palmitoleic acid), carnitines (carnitine, hexanoylcarnitine and propionylcarnitine) and others (adenosine, bilirubin, cortisol, hippuric acid, hypoxanthine, lactic acid, oleamide and urea).

**Figure 1:**
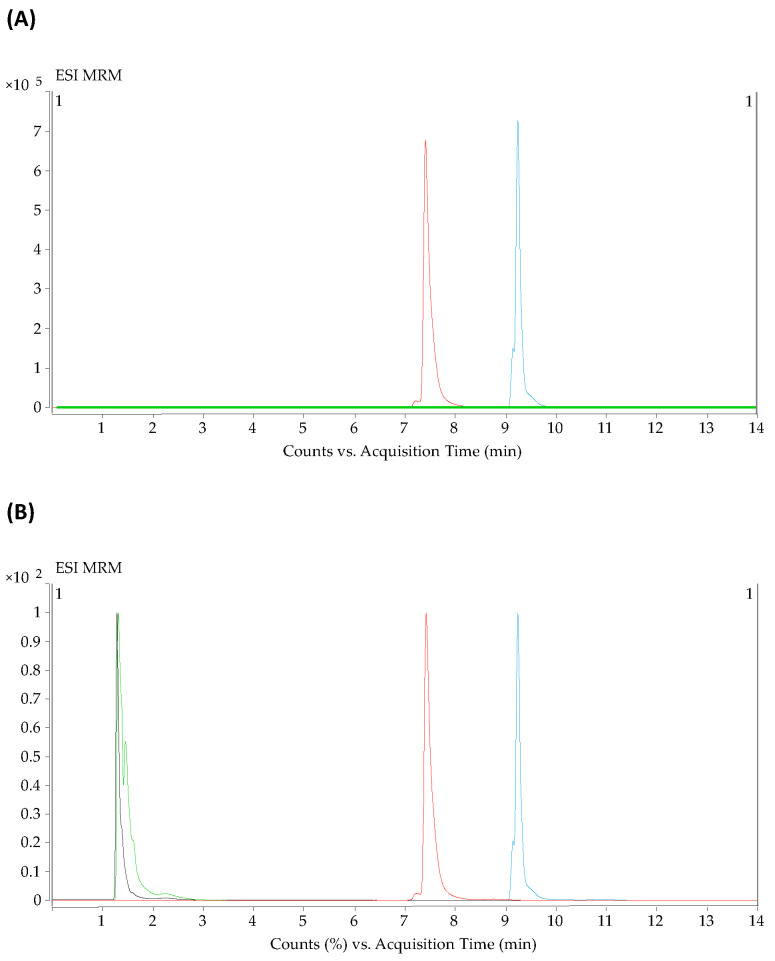
The effect of mobile phases modifier on fatty acid. (**A**) Chromatographic profile of LPC 16:0 (red) and LPE 18:0 (blue) using ammonium formate as modifier. Oleic (black) and lauric acid (green) showing zero abundance. (**B**) Chromatographic profile of LPC 16:0 (red) and LPE 18:0 (blue) using ammonium acetate as modifier.

**Figure 2:**
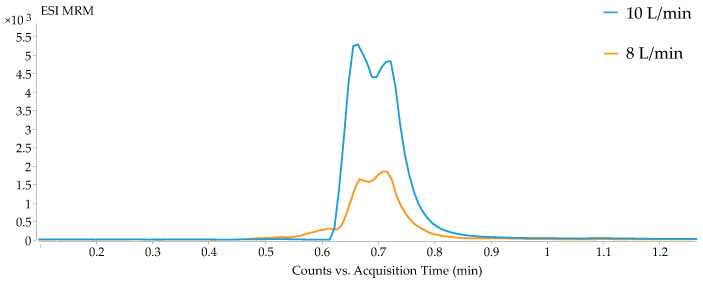
Comparative abundance of lactic acid using a gas and sheath gas flow of 8 L/min (orange) or 10 L/min (blue).

The development of a single method to analyze different classes of metabolites, all with different concentrations in the sample, is a tremendous challenge. The first step in this approach entailed dividing the metabolites into two groups, according to their physicochemical properties, each to be subjected to a different analytical method. On the one hand, the molecules capable of forming hydrophilic interactions were selected for the hydrophilic interaction liquid chromatography (HILIC) method to be retained, while the molecules with moderate polarity were assigned for the reversed-phase method. As the selected metabolites ranged at different concentrations in the samples, serial dilutions were tested (1/2, 1/4, 1/8, 1/16, 1/32, 1/64 and 1/128) for the extracted supernatant. After studying the compromise between analytical signal (saturation and detection) and dilution (the greater the dilution, the lower the matrix effect observed), the 1/8 dilution was selected as optimal, as it had a good signal detection for all metabolites, and linearity was not lost by saturation of the detector. Additionally, after an extensive search of chromatographic conditions for each metabolite class, we decided to start with formic acid, which is the most common modifier for the mobile phases in reversed-phase chromatography [26]. However, we observed that a group of metabolites, namely the fatty acids (palmitoleic acid, oleic acids, lauric acid, and arachidonic acids), did not ionize with this modifier. To address this problem, acetic acid was used, and the fatty acids were then properly detected (Figure 1). An additional problem to be solved was that the lactic acid, which eluted before the first minute, when 100% of the mobile phase gradient was aqueous, was not correctly ionized and their abundance was quite low. We, therefore, decided to increase both the gas flow and sheath gas flow from 8 to 10 L/min to improve their ionization, obtaining an increased abundance from 1.5 × 10^3^ to 5.3 × 10^3^ counts (Figure 2).

In the case of HILIC, ammonium acetate was used as a typical modifier for this type of chromatography [27]. Therefore, the pH was adjusted below the pKa values of the carnitines by adding 0.1% of acetic acid, reaching around pH = 3.3. We, thus, ensured that all carnitines were detected as the pKa values are 4.22 for hexanoylcarnitine, 4.19 for propionylcarnitine and 3.8 for carnitine. For sample preparation, the extracted supernatant was diluted in the initial mobile phases of HILIC, as this method achieves maximum reproducibility. In addition, this does not affect the reproducibility of the reversed-phase chromatography. To optimize the time spent in the sample treatment, samples were prepared once and analyzed by these two complementary chromatographic methods. Finally, metabolite transitions were optimized for HILIC and reversed-phase methods (Table 1). A representative chromatogram of each method is shown in Figures S1 and S2, respectively.

### 2.2. Validation Study

The parameters evaluated in the validation study corresponded to the linearity of the method, selectivity, precision of the method, and recovery. Selectivity was assessed by observing only the signals in the standards but none in the blank injections for most metabolites. Method linearity revealed a linear regression coefficient (*r*) that ranged from 0.99 to 1.00 and 0.97 to 1.00 for the standard and sample, respectively, considering both HILIC and reversed-phase methods (Table 2). However, the use of mass spectrometry (MS) is often linked to the matrix effect in the metabolite determinations, compromising both method precision and selectivity [28,29]; therefore, we tested our methodology for this effect. Table 2 shows that independently of the analytical method employed, all metabolites showed a matrix effect after obtaining a significant *p*-value (*p* < 0.05), when comparing statistically the slopes of the standards and standard addition calibration curve.

In the case of method precision and recovery (Table 3), the relative standard deviation (RSD) in the HILIC method ranged from 1.88% to 6.99% for intra-day analysis (*n* = 6) and 0.4% to 17.17% for inter-day analysis (*n* = 12) and a recovery range from 81.42% to 118.02%. For the metabolites analyzed by the reversed-phase method, the precision ranged from 0.47% to 13.67% for intra-day (*n* = 6) and from 0.79% to 27.64% for inter-day analysis. In addition, their recovery ranged from 79.31% to 116.24%, except for oleamide (33.98%), LPI 20:4 (50.33%), and SPA-1P (132.39%).

### 2.3. Quantification of Metabolites in a Pooled Serum Sample

As we were proposing a new quantification procedure, we decided to test the accuracy of this approach with those values reported in the literature in online data bases, such as the Human Metabolome Database (HMDB). To do this, a pooled serum sample was analyzed with our methodology, and metabolite concentrations were compared with values from healthy adults published in the HMDB (Table 4). We found values reported for 29 out of 36 metabolites for healthy subjects in serum. Of these, 15 were within the range published in HMBD (>50%). To our knowledge, none of these metabolites have been reported in asthma or any allergic condition in the HMDB database.

### 2.4. Clinical Models

#### 2.4.1. Metabolite Quantification

Regarding the quantification of this set of metabolites in the serum samples, we confirmed that all relative areas were within the range studied for the validation.

#### 2.4.2. Clinical Characteristics of Patients

Regarding the application of this methodology, the following two independent models were studied depending on the sex of the patients: (1) ICS vs. UC groups (both with female patients) and (2) IT vs. BIO groups (both with male and female patients).

The clinical history of the patients was studied thoroughly, and no differences were found in relation to body mass index (BMI), onset age, smoking habits, or total IgE levels among the groups of the clinical models (*p* > 0.05) (Table 5). Additionally, age was only significant between the IT and BIO groups (*p* < 0.05).

With regard to medication, patients from the ICS group were treated with antihistamines (AH), topical corticosteroids (Topic CS), and inhaled CS with a long-acting beta-adrenoceptor agonist (inhaled CS/LABA). Moreover, one of these patients was prescribed singulair (S) and the other one short-effect bronchodilators (SABA). Regarding UC patients, all were treated with inhaled CS/LABA and SABA, 50% of them were treated with AH and Topic CS, 75% with S and, 25% with anticholinergics (AC) and theophylline (T). In the case of BIO patients, a similar treatment as in the UC group is applied, with the exception that no BIO patient was treated with T. Finally, the IT medication pattern resembles that of the ICS group, although only 6 patients out of 23 were taking inhaled CS/LABA, while most of them (78.26%) were prescribed SABA. Individual clinical characteristics of patients are shown in Table S2.

#### 2.4.3. ICS versus UC

**Figure 3:**
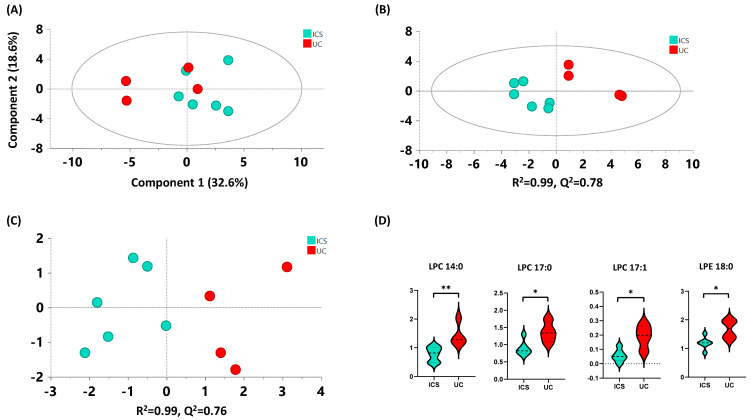
(**A**) PCA model, (**B**) PLS-DA model, and (**C**) CV-OPLS-DA model for ICS vs. UC comparison. Data were UV scaled. R^2^ is the capability of the model to classify the samples; Q^2^ is the capability of the model to predict the class of a new simple. (**D**) Significant metabolites between the ICS and UC groups. (Key: “ICS”: blue, “UC”: red). * *p* < 0.05; ** *p* < 0.01.

As a starting point, 36 quantified metabolites were analyzed using multivariate analysis to explore if there was any trend of clustering towards the study groups. A total of five metabolites (LPC 19:0, LPI 16:0, LPI 20:4, oleamide, and SPA-1P) were excluded from the analysis since their abundance was below 10^2^ counts. A non-supervised principal component analysis (PCA) model showed a tendency to separate both studied groups by the first component with 32.6% of explained variability in the first component (Figure 3A). Moreover, to evaluate the differences between these groups, a partial least squares-discriminant analysis (PLS-DA) model was performed (Figure 3B). Interestingly, this model showed a clear separation of the groups obtaining good quality parameters (R^2^ = 0.99 and Q^2^ = 0.78). Finally, to determine the differences exclusively related to the classification an orthogonal (O)PLS-DA model was performed, showing good parameters (R^2^ = 0.99 and Q^2^ = 0.76), and the cross validated-scores (CV-scores) plot showed that 100% of the samples were correctly classified into their corresponding clinical group (Figure 3C). In addition, univariate statistical analyses were performed and showed differences in the metabolic signature of UC versus ICS. A total of 4 metabolites out of 31 showed significant differences (*p* < 0.05) between groups (LPE 18:0, LPC 17:0, LPC 14:0 and LPC 17:1), in that all were increased in UC versus ICS (Figure 3D and Table S3).

In summary, with the metabolites from our targeted method, the multivariate models were able to distinguish both groups.

#### 2.4.4. IT versus BIO

**Figure 4:**
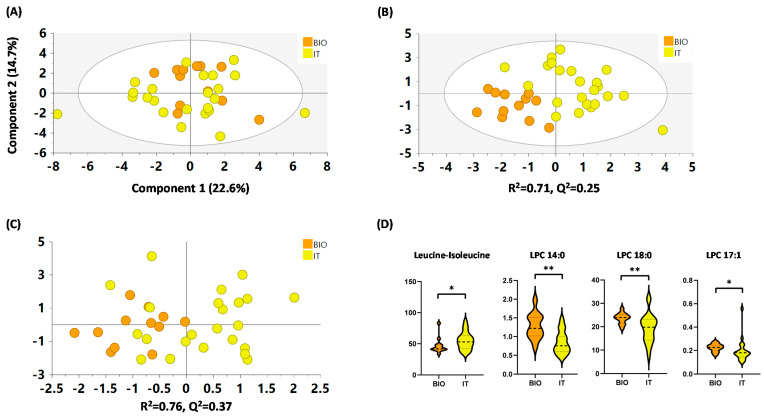
(**A**) PCA model, (**B**) PLS-DA model, and (**C**) CV-OPLS-DA model for IT vs. BIO comparison. Data were UV scaled. R^2^ is the capability of the model to classify the samples; Q^2^ is the capability of the model to predict the class of a new sample. (**D**) Comparison of significant metabolites by univariate statistics between the BIO and IT groups. Key: “BIO”: orange, “IT”: yellow. * *p* < 0.05; ** *p* < 0.01.

In the case of the IT and BIO groups, comparisons between the groups were performed following the same approach as the ICS vs. UC comparison. In this analysis, a total of 8 metabolites out of the 36 quantified were excluded because their abundance was below 10^2^ counts (lauric acid, LPC 16:0, LPC 19:0, LPI 20:4, oleamide, SPA-1P, sphinganine-C17 and sphingosine). In the PCA model, multivariate analysis showed no clear trend for the groups (Figure 4A). However, the supervised PLS-DA model showed a good classification parameter (R^2^ = 0.71), and the CV-scores of the OPLS-DA showed a 100% correct classification of the BIO samples and almost 70% of the IT samples (Figure 4B,C, respectively). Furthermore, the univariate analysis showed that 4 metabolites out of 28 had significant differences (*p* < 0.05) between groups (leucine/isoleucine, LPC 18:0, LPC 14:0 and LPC 17:1); all LPCs increased in BIO and leucine/isoleucine increased in IT in comparison to the other group (Figure 4D and Table S3).

To sum up, with this set of metabolites, we were able to observe differences between the groups and to correctly classify all the BIO samples.

## 3. Discussion

Only a few non-targeted metabolomic studies include a targeted analysis of the findings. In the ones that do, this is usually based on a specific biochemical class to simplify the analytical method. The development of a method that includes different biochemical classes with different physico-chemical properties and concentration levels is an arduous task. In addition, the study of allergies and their complications, such as allergic asthma and in the worst-case anaphylaxis, makes this even harder to study. To our knowledge, this is the first time that three non-targeted metabolomics studies have been used to elaborate a targeted method to select those significant metabolites with available commercial standards.

In the case of our method development, it was important to consider the chromatography selected and its requirements; in other words, the sample dilution required to achieve the best compromise between detection and matrix effect, the modifier and the pH of the mobile phases, and the ion source parameters. These parameters or others must be optimized in accordance with the metabolites of interest.

Regarding method validation, we observed that all metabolites followed method linearity, observing *r* > 0.97. Surprisingly, all metabolites also presented a matrix effect. This behavior is usually overcome by employing at least one stable isotope-labeled internal standard for each metabolite class [30], although often these are not available. We, therefore, propose a new approach for metabolite quantification based on the preparation of external calibration curves using the extracted sample. With this approach, the standards in the calibration curve follow the same matrix effect as the samples. We also verified the precision and recovery for most of the metabolites, meaning that these could be quantified as described previously. On the contrary, for metabolites with a recovery <75%, we proposed the use of a correction factor. With this strategy, we were able to study the capabilities of our methodology and address its matrix effect and recovery. This was proved by comparing the values with those published in HMDB for healthy adults.

This methodology was applied to study patients with allergic asthma. The patient cohort was divided into the following two clinical models: (1) female patients controlled with ICS compared to female UC patients, and (2) patients controlled with IT versus patients with BIO (both with female and male patients). Regarding the female ICS versus female UC, we observed a clear separation of the groups using non-supervised and supervised models, indicating that these metabolites were able to differentiate both groups. The increase in LPE 18:0, LPC 17:0, LPC 14:0, and LPC 17:1 in the UC group followed the same trend as those observed previously in the non-targeted analysis [16,17,18]. Regarding these changes, LPCs have previously been associated with inflammatory signaling pathways in asthma, rhinitis, and eosinophilic asthma [31,32,33]. Furthermore, it is important to consider the relation between gut microbiota and the host immune system in allergic disorders [23,34] and the fact that increased levels of odd-chain fatty acids, such as LPC 17:0 and LPC 17:1, have been shown to decrease the risk of cardiovascular disease [35] and allergy in offspring [36]. However, to our knowledge, this is the first time that increased levels of these fatty acids have been related to a severe asthmatic phenotype. Thus, further studies will be needed to better clarify the role of odd-chain fatty acids in asthma phenotypes. In addition, several glycerophospholipids, including LPE (16:1), have been linked to chronic obstructive pulmonary disease, showing anti-inflammatory activity [37]. However, further studies are needed for other LPEs, such as LPE 18:0, to better characterize their role in this phenotype.

In the case of comparison of IT versus BIO, due to the heterogeneity of the IT group, and the fact that not all patients have been treated with the same type of IT and for the same length of time, the difference between the groups was not as clear as for model 1. Furthermore, it is estimated that IT is successful in around 70% of cases. The increase in LPCs (LPC 14:0, LPC 17:1, and LPC 18:0), as with the previous comparison, in the BIO group compared to IT reinforces the idea that LPCs act as proinflammatory mediators in allergic airway diseases [38]. Finally, in the case of leucine/isoleucine, which was increased in the IT group compared to BIO, it has been described to regulate the mammalian target of rapamycin (mTOR) activation, which has an important role in house dust mite-induced allergic asthma, through the regulation of T lymphocyte cell proliferation and differentiation [39].

This pilot study has some limitations. The number of samples from the clinical models is not very large and a larger cohort of samples is required to validate the results obtained. Moreover, a more complete clinical history is needed to explain why some IT samples do not classify within their group.

## 4. Materials and Methods

### 4.1. Chemical and Reagents

Reverse-osmosed ultrapure water was obtained from a Milli-Q Plus185 system (Millipore, Billerica, MA, USA). A MS grade methanol (MeOH), used for standards preparation, and acetonitrile (ACN) were obtained from Fisher Scientific (Hampton, NH, USA). Analytical grade ammonia solution (28%, GPR RECTAPUR^®^) and acetic acid glacial (AnalaR^®^ NORMAPUR^®^) were obtained from VWR Chemicals (Radnor, PA, USA).

Arachidonic acid, LPC 14:0, LPC 16:0, LPC 17:0, LPC 17:1, LPC 18:0, LPC 18:1, LPC 19:0, LPE 18:0, LPI 16:0, LPI 20:4, palmitoleic acid, oleamide, SPA-1P, sphinganine-C17, sphingosine, S1P, sphingosine d7, LPC 18:1 d7 were purchased from Avanti lipids (Birmingham, AL, USA). Bilirubin, lactic acid, lauric acid, oleic acid, adenosine, arginine, betaine, carnitine, cortisol, creatine, creatinine, hexanoylcarnitine, hippuric acid, hypoxanthine, leucine/isoleucine, phenylalanine, proline, propionylcarnitine, urea, carnitine d3, isoleucine d7, and palmitic acid d31 were purchased from Sigma-Aldrich (Darmstadt, Germany). Phenylalanine d5 and valine d8 were purchased from Cambridge Isotope Laboratories Inc. (Andover, MA, USA).

### 4.2. HPLC-QqQ-MS Analytical Methods

***Instrumentation.*** Samples were measured using dynamic molecular reaction monitoring (dMRM) on a liquid chromatography (LC) system (1260 Infinity, Agilent Technologies, Santa Clara, CA, USA), coupled to a triple quadrupole mass spectrometer with electrospray ionization Agilent Jet Stream source (ESI (AJS)-QqQ-MS), 6470 Agilent Technologies (Santa Clara, CA, USA).

Standard calibration curves, internal standards (ISTD) and samples were prepared in the same way for two complementary methods to minimize time consumption. The first was the HILIC method, which was used to detect small polar metabolites (adenosine, betaine, carnitine d3, cortisol, creatine, creatinine, hexanoylcarnitine, hippuric acid hypoxanthine, isoleucine d7, arginine, carnitine, leucine/isoleucine, phenylalanine, phenylalanine d5, proline, propionylcarnitine, urea, and valine d8), mainly amino acids and short-chain acids. The second one was the reversed-phase method, which was applied to detect metabolites with medium polarity (arachidonic acid, bilirubin, lactic acid, lauric acid, LPC 14:0, LPC 16:0, LPC 17:0, LPC 17:1, LPC 18:0, LPC 18:1, LPC 18:1 d7, LPC 19:0, LPC 20:0, LPE 18:0, LPI 16:0, LPI 20:4, oleamide, oleic acid, palmitic acid d31, palmitoleic acid, SPA-1P, sphinganine-C17, sphingosine, sphingosine d7, and S1P), mainly phospholipids and sphingolipids.

***HILIC method.*** Metabolite separation was achieved using gradient elution on a Kinetex HILIC (150 mm × 2.1 mm × 100 Å) column maintained at 25 °C. The mobile phases consisted of (A) water, and (B) ACN, both with 7.5 mM ammonium acetate and 0.1% acetic acid, obtaining a final pH of 4.0 in the aqueous phase. The flow rate was 0.5 mL/min. The gradient started with 5% of A for 2 min, then increased up to 50% until 12 min, and back to initial conditions until 22 min. The MS conditions were as follows: 5500 V of capillary voltage in positive ESI mode, a nebulizer gas flow rate of 11.0 L/min, a source temperature of 250 °C; and a source pressure of 60 psi.

***Reversed-phase method***. Metabolite separation was achieved using gradient elution on a Supelco Ascentis Express reversed-phase (150 mm × 2.1 mm × 2.7 μm) column maintained at 60 °C. The mobile phases consisted of (A) water, and (B) ACN, both with 0.1% acetic acid, obtaining a final pH of 3.3 in the aqueous phase. The flow rate was 0.6 mL/min. The gradient started with 20% of B for 2 min, then increased up to 100% until 10 min and maintained for 5 min, then returned to initial conditions until 20 min. The MS conditions were as follows: 3500 V of capillary voltage in positive ESI mode and 3000 V in negative ESI mode, a nebulizer gas flow rate of 10.0 L/min, a source temperature of 250 °C; and a source pressure of 45 psi.

For both methods, LC vials were maintained at 4 °C in a thermostatic autosampler, and the injection volume was set at 5 µL.

### 4.3. Sample Preparation

**Figure 5:**
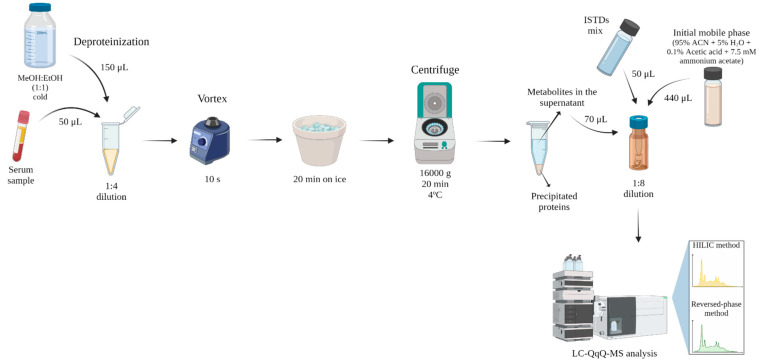
Scheme of serum sample preparation. *ACN*: acetonitrile; *ISTDs*: internal standards; *MeOH:EtOH*: methanol:ethanol.

Samples were prepared by mixing 50 µL of serum with 150 µL of cold (−20 °C) methanol: ethanol mix (MeOH:EtOH) (1:1), diluting the serum 4 times. Samples were then vortex-mixed for 1 min, kept on ice for 15 min and centrifuged at 16,000× *g* for 20 min at 4 °C. Then, 70 µL of the supernatant were transferred into an LC vial and mixed with 50 µL of ISTD mix, (with a final concentration of 0.2 µg/mL for HILIC and 0.3 µg/mL for reversed-phase method) and 440 µL of the initial conditions of the mobile phases of the HILIC method for the analysis, diluting the supernatant 8 times (Figure 5). For blank preparation, 50 µL of water was used and the same steps as for the samples were followed.

### 4.4. Metabolite Quantification

Considering that these metabolites are endogenous and considering the matrix effect observed, an external calibration curve was prepared using equal volumes 1:1 (*v:v*) of a pool of extracted serum samples (dilution 1:8) and each of the dilutions (0%, 25%, 50%, 100%, 200%, 400% and 800%) that were prepared for the standard calibration curve containing the ISTD and external standards. Two independent external calibration curves were prepared to contain the specific standards for each chromatographic method. The order of the worklist for sample analysis was as follows: 3 blank injections, 10 injections to equilibrate the column with a quality control prepared by a pool of equal volumes of all the study serum samples, the 7 levels of the calibration curve (from the most diluted to the most concentrated one), a group of samples injected in a random order, and again the calibration curve in a way that was analyzed at least once per day of analysis. The worklist ended with another 3 blank injections.

**Figure 6:**
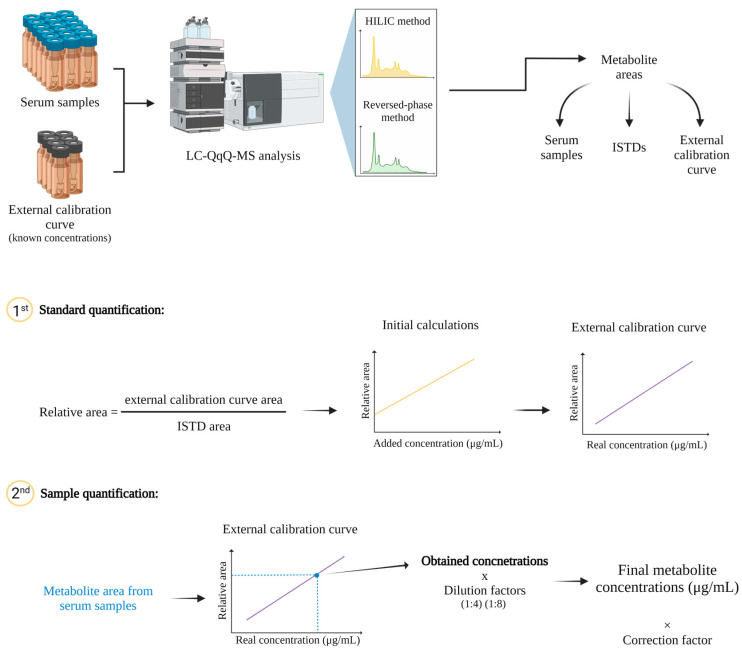
Diagram of metabolite quantification.

The external calibration curves were generated by plotting on the Y-axis the peak relative area (area of the metabolite/area of the corresponding ISTD) versus the X-axis with the estimated concentrations (concentration of the pool of extracted serum samples plus the added standard). The metabolites with acceptable linearity and recovery from validation were quantified by the interpolation in their corresponding external calibration curve. Final concentrations (µg/mL) were calculated considering the sample dilutions. Metabolites that did not achieve an acceptable recovery were estimated by applying a correction factor calculated by the ratio between 100% recovery and the recovery percentage obtained from 12 independent replicates (Figure 6).

### 4.5. Data Treatment

Chromatogram visualization and peak areas were obtained using MassHunter Workstation B.05.00 and MassHunter QQQ Quantitative Analysis B.08.00 (Agilent Technologies, Santa Clara, CA, USA), respectively.

### 4.6. Method Development

Chromatographic conditions, such as the modifier (ammonium formate and ammonium acetate) and pH adjustment, were tested for the development of both models. Regarding MS conditions, gas flow and sheath gas flow were adjusted to increase sensitivity for some metabolites.

Finally, the transitions, fragmentor and collision energy voltages were optimized for both the HILIC and the reversed-phase methods using the MassHunter Workstation Optimizer 10.0.127 (Agilent Technologies, Santa Clara, CA, USA). The optimizer used collision energy ranging from 5 to 50 eV and selected a precursor ion of H^+^ for positive ions and H^−^ and CH_3_COO^−^ for negative ions. Additionally, the fragmentor range was established from 40 to 200 eV and 4 product ions and a low mass cut of 20 *m*/*z* were considered.

### 4.7. Method Validation

Both methods were validated in terms of selectivity, linearity of the standard and sample, matrix effect, recovery, and intra-day and inter-day precision following the ICH harmonized guidelines [40]. For the validation, individual standards were prepared at 1000 µg/mL by weighing the reagent and dissolving it in their corresponding solvent. Then, for linearity, a stock-mix standard solution was prepared for each method by mixing a volume of their corresponding metabolites 10 times more concentrated than a previously estimated pool of serum samples, for which the levels were considered 100%. From these, several dilutions were prepared (25%, 50%, 100%, 200%, 400% and 800%) to generate the standard calibration levels. The corresponding ISTD mix was added to each of these to reach a final concentration of 0.2 µg/mL and 0.3 µg/mL for HILIC and reversed-phase methods, respectively. For sample linearity, a standard addition calibration curve was prepared. Method linearity (sample and standard) was evaluated by triplicate. Selectivity of metabolites and ISTD were tested by the injection of three blank samples and three 100% of stock-mix standard solutions for each method.

The matrix effect was examined by comparing the slopes of curves (linearity of standard and sample) using a *t*-test, considering a *p* < 0.05 as reflecting the existence of a matrix effect for the respective metabolite. Regarding recovery, the 100% of standard addition calibration curve level was analyzed six times per day on two different days (in total *n* = 12). The concentration was calculated by the interpolation of each metabolite in the external calibration curve described in Section 2.4. Finally, recovery was obtained by comparing the concentrations of the one obtained experimentally, and the theoretical calculated concentration expressed as a percentage (%). The precision of the method was determined by calculating the RSD of the relative area. For both recovery and precision, the intra-days (*n* = 6) and inter-days (*n* = 12) were examined.

### 4.8. Clinical Models and Sample Collection

Serum samples from allergic asthma patients were obtained in the Allergy Service at Hospital Universitario de Gran Canaria Doctor Negrín (Las Palmas de Gran Canaria, Spain). All patients signed informed consent and the study was approved by the Ethics Committee of the hospital on the 4 February 2016 (code: 160009).

The patient cohort was divided into the following four groups according to their response to treatment: going from corticosteroid-controlled (ICS, *n* = 6), immunotherapy-controlled (IT, *n* = 23), biological-controlled (BIO, *n* = 12) and uncontrolled (UC, *n* = 4) patients. After looking at the sex of the groups, two clinical models were chosen. These included clinical model 1, where all the patients were female and the ICS was compared with the UC group, and clinical model 2, where patients of both sexes with different pharmacological treatment IT versus BIO were compared (Table S2).

For sample collection, whole blood was taken and incubated with a clotting agent using Vacutainer SST II tubes. Samples were placed at room temperature for 30 min and then centrifuged at 2000× *g* for 10 min. Serum was recovered and stored at −80 °C until the metabolomic analysis was performed.

### 4.9. Statistical Analysis of the Clinical Models

The differences between groups were investigated using the Mann–Whitney U test with a Benjamini–Hochberg (BH) *p*-value correction, also known as false discovery rate (FDR). Univariate statistical analyses were performed using Matlab R2018b (Mathworks) software and the statistical significance was set at a 95% level (*p* < 0.05). Multivariate analysis was performed using SIMC A P + 16.0 (Sartorius Stedim Data Analytics AB, Umeå, Sweden). The PCA model was applied to observe data patterns, while the PLS-DA model was used to evaluate metabolite differences between groups, and the OPLS-DA model was used to assess the separation of the two groups on the X-axis. The robustness of the models was evaluated based on R^2^ (explained variance) and Q^2^ (capability of prediction) scores [41,42]. Raw data treatment and graphics were performed using Excel 2016 (Microsoft, Redmond, WA, USA) and GraphPad Prism v8.1.2 (San Diego, CA, USA), respectively.

## 5. Conclusions

To sum up, we have developed a new targeted methodology for the analysis of metabolites selected from three non-targeted metabolomic analyses for studying severe allergic phenotypes. This methodology comprising 36 metabolites (corresponding mainly to phospholipid, carnitines, and sphingolipid pathways, energy metabolism, and amino acids) measured in two chromatographic methods can be of great importance to further understand their role in allergic diseases. These metabolites can also be potential biomarkers for the diagnosis of disease severity and can be used to predict the patient response to a treatment such as IT and BIO, and in the future, could help with exploring new pharmacological allergy treatments.

## Figures and Tables

**Table 1:** Optimized metabolite parameters for HILIC and reversed-phase methods.

dMRM									
Metabolite	Precursor Ion (*m/z*)	Product Ion (*m/z*)	Transition Type	Ret Time (min)	Delta Ret Time (min)	Fragmentor (eV)	CE (eV)	CAV (eV)	ESI Mode
**HILIC Method**									
Adenosine	268.11	135.90	Quantifier	3.02	5	100	20	7	Positive
Adenosine	268.11	118.90	Qualifier	3.02	5	100	56	7	Positive
Arginine	175.12	70.10	Quantifier	12.41	5	89	29	7	Positive
Arginine	175.12	60.10	Qualifier	12.41	5	89	13	7	Positive
Betaine	118.09	58.00	Quantifier	9.90	5	100	36	7	Positive
Betaine	118.09	42.00	Qualifier	9.90	5	100	72	7	Positive
Carnitine	162.11	85.00	Qualifier	11.80	5	80	19	7	Positive
Carnitine	162.11	60.10	Quantifier	11.80	5	80	15	7	Positive
Carnitine d3	165.13	63.00	Qualifier	11.79	5	100	20	7	Positive
Carnitine d3	165.13	43.00	Quantifier	11.79	5	100	36	7	Positive
Cortisol	363.22	121.00	Qualifier	0.91	5	121	25	7	Positive
Cortisol	363.22	91.00	Quantifier	0.91	5	121	77	7	Positive
Creatine	132.08	44.20	Quantifier	9.81	5	99	25	7	Positive
Creatine	132.08	43.10	Qualifier	9.81	5	99	57	7	Positive
Creatinine	114.07	44.20	Quantifier	6.45	5	104	17	7	Positive
Creatinine	114.07	43.10	Qualifier	6.45	5	104	57	7	Positive
Hexanoylcarnitine	260.19	84.90	Quantifier	9.99	5	100	24	7	Positive
Hexanoylcarnitine	260.19	29.10	Qualifier	9.99	5	100	64	7	Positive
Hippuric acid	180.07	105.00	Quantifier	6.54	5	72	13	7	Positive
Hippuric acid	180.07	77.00	Qualifier	6.54	5	72	37	7	Positive
Hypoxanthine	137.05	118.90	Qualifier	3.01	5	100	24	7	Positive
Hypoxanthine	137.05	55.00	Quantifier	3.01	5	100	36	7	Positive
Isoleucine d7	139.16	92.00	Quantifier	8.86	5	100	8	7	Positive
Isoleucine d7	139.16	47.00	Qualifier	8.86	5	100	28	7	Positive
Leucine/isoleucine	132.10	86.00	Quantifier	8.64	5	100	8	7	Positive
Leucine/isoleucine	132.10	30.10	Qualifier	8.64	5	100	20	7	Positive
Phenylalanine	166.09	119.90	Quantifier	8.33	5	100	16	7	Positive
Phenylalanine	166.09	76.90	Qualifier	8.33	5	100	48	7	Positive
Phenylalanine d5	171.12	124.90	Quantifier	8.4	5	131	16	7	Positive
Proline	116.07	70.00	Quantifier	9.47	5	100	20	7	Positive
Proline	116.07	28.10	Qualifier	9.47	5	100	48	7	Positive
Propionylcarnitine	218.14	84.90	Quantifier	11.19	5	100	20	7	Positive
Propionylcarnitine	218.14	29.10	Qualifier	11.19	5	100	52	7	Positive
Urea	61.04	44.00	Quantifier	1.90	5	100	24	7	Positive
Urea	61.04	29.10	Qualifier	1.90	5	100	90	7	Positive
Valine d8	126.14	80.10	Quantifier	9.64	5	67	13	7	Positive
**Reversed-phase method**									
Arachidonic acid	303.20	303.20	Quantifier	9.86	6	131	0	7	Negative
Arachidonic acid	303.20	259.20	Qualifier	9.86	6	131	13	7	Negative
Bilirubin	585.27	299.10	Quantifier	10.11	6	131	25	7	Positive
Bilirubin	585.27	271.10	Qualifier	10.11	6	131	50	7	Positive
Lactic acid	89.00	89.00	Quantifier	1.00	6	45	0	7	Negative
Lactic acid	89.00	43.10	Qualifier	1.00	6	45	9	7	Negative
Lauric acid	199.20	199.20	Quantifier	8.74	6	45	0	7	Negative
LPC 14:0	468.31	183.90	Quantifier	7.83	6	100	28	7	Positive
LPC 14:0	468.31	103.90	Qualifier	7.83	6	100	56	7	Positive
LPC 16:0	496.34	183.80	Quantifier	8.63	6	100	28	7	Positive
LPC 16:0	496.34	103.90	Qualifier	8.63	6	100	28	7	Positive
LPC 17:0	510.36	184.00	Quantifier	9.06	6	180	29	7	Positive
LPC 17:0	510.36	104.00	Qualifier	9.06	6	180	29	7	Positive
LPC 17:1	508.34	183.90	Quantifier	8.45	6	185	29	7	Positive
LPC 17:1	508.34	104.00	Qualifier	8.45	6	185	29	7	Positive
LPC 18:0	524.37	183.80	Quantifier	9.55	6	100	28	7	Positive
LPC 18:0	524.37	103.90	Qualifier	9.55	6	100	28	7	Positive
LPC 18:1	522.36	183.80	Quantifier	8.85	6	100	28	7	Positive
LPC 18:1	522.36	103.90	Qualifier	8.85	6	100	28	7	Positive
LPC 18:1 d7	529.40	183.80	Quantifier	9.76	6	100	32	7	Positive
LPC 18:1 d7	529.40	103.90	Qualifier	9.76	6	100	28	7	Positive
LPC 19:0	539.39	183.80	Quantifier	10.48	6	100	32	7	Positive
LPC 19:0	538.39	183.90	Qualifier	10.48	6	100	28	7	Positive
LPE 18:0	482.33	341.10	Qualifier	8.90	6	100	20	7	Positive
LPE 18:0	482.33	44.10	Quantifier	8.90	6	100	20	7	Positive
LPI 16:0	573.30	555.20	Qualifier	9.83	6	104	5	7	Positive
LPI 16:0	573.30	313.20	Quantifier	9.83	6	104	25	7	Positive
LPI 20:4	621.31	603.30	Qualifier	9.77	6	89	5	7	Positive
LPI 20:4	621.31	361.30	Quantifier	9.77	6	89	13	7	Positive
Oleamide	282.28	55.10	Qualifier	9.84	6	104	45	7	Positive
Oleamide	282.28	41.20	Quantifier	9.84	6	104	69	7	Positive
Oleic acid	281.25	281.25	Quantifier	9.50	6	161	0	7	Negative
Palmitic acid d31	286.23	286.23	Quantifier	10.13	6	156	0	7	Negative
Palmitoleic acid	253.23	253.23	Quantifier	9.66	6	109	0	7	Negative
SPA-1P	382.27	382.27	Qualifier	7.79	6	130	0	7	Positive
SPA-1P	382.27	284.10	Quantifier	7.79	6	130	12	7	Positive
SPA-1P	382.27	60.00	Qualifier	7.79	6	130	24	7	Positive
Sphinganine-C17	288.29	288.29	Quantifier	6.53	6	100	0	7	Positive
Sphinganine-C17	288.29	60.00	Qualifier	6.53	6	100	12	7	Positive
Sphinganine-C17	288.29	30.10	Qualifier	6.53	6	100	80	7	Positive
Sphingosine	300.29	282.10	Quantifier	6.59	6	100	8	7	Positive
Sphingosine	300.29	55.40	Qualifier	6.59	6	100	40	7	Positive
S1P	380.26	264.00	Quantifier	7.62	6	100	16	7	Positive
S1P	380.26	81.90	Qualifier	7.62	6	100	36	7	Positive
Sphingosine d7	307.34	289.10	Quantifier	6.72	6	100	12	7	Positive
Sphingosine d7	307.34	30.10	Qualifier	6.72	6	100	84	7	Positive

CAV: collision cell accelerator voltage; CE: collision energy; dMRM: dynamic multiple reaction monitoring; ESI: electrospray ionization; LPC: lysophosphocholine; LPE: lysophosphoethanolamine; LPI: lysophosphatidylinositol; Ret: retention; SP1: sphingosine-1-phosphate; SPA-1P: sphinganine-1-phosphate.

**Table 2:** Validation parameters for HILIC and reversed-phase method.

Metabolite	ISTD Used	Blank Signal (%) ^&^	Standard Linearity (*r*)	Sample Linearity (*r*)	Matrix Effect (*p*-Value)	External Calibration Curve Range (µg/mL)
**HILIC Method**						
Adenosine	Carnitine d3	3.01	0.992	0.987	<0.05	0.000–0.001
Arginine	Carnitine d3	4.51	0.993	0.995	<0.05	0.325–3.273
Betaine	Carnitine d3	37.95	0.990	0.996	<0.05	0.282–1.028
Carnitine	Carnitine d3	0.85	1.000	0.999	<0.05	0.195–1.643
Cortisol	Carnitine d3	NA	0.994	0.998	<0.05	0.005–0.109
Creatine	Carnitine d3	0.33	0.989	0.998	<0.05	0.337–1.876
Creatinine	Carnitine d3	0.02	0.995	0.998	<0.05	0.400–1.256
Hexanoylcarnitine	Carnitine d3	0.41	0.994	0.997	<0.05	0.000–0.003
Hippuric acid	Carnitine d3	0.02	0.992	0.998	<0.05	0.010–0.099
Hypoxanthine	Carnitine d3	1.00	0.997	0.999	<0.05	0.027–0.501
Leucine/isoleucine	Isoleucine d7	NA	0.999	0.999	<0.05	0.570–3.599
Phenylalanine	Isoleucine d7	0.04	0.998	0.999	<0.05	0.163–2.289
Proline	Isoleucine d7	0.53	0.999	0.994	<0.05	1.003–3.422
Propionylcarnitine	Carnitine d3	0.16	0.992	0.996	<0.05	0.001–0.028
Urea	Carnitine d3	0.01	0.998	0.996	<0.05	11.40–36.41
**Reversed-phase method**						
Arachidonic acid	Palmitic acid d13	NA	0.995	0.996	<0.05	0.034–0.245
Bilirubin	Palmitic acid d13	NA	0.994	0.977	<0.05	0.551–3.623
Lactic acid	Palmitic acid d13	0.58	0.991	0.987	<0.05	9.898–26.732
Lauric acid	Palmitic acid d13	48.27	0.998	0.992	<0.05	0.062–0.434
LPC 14:0	LPC 18:1 d7	0.65	0.999	0.999	<0.05	0.0018–0.196
LPC 16:0	LPC 18:1 d7	0.69	0.999	1.000	<0.05	1.436–7.038
LPC 17:0	LPC 18:1 d7	NA	0.999	0.999	<0.05	0.020–0.193
LPC 17:1	LPC 18:1 d7	37.94	0.999	0.998	<0.05	0.001–0.038
LPC 18:0	LPC 18:1 d7	1.29	0.999	0.999	<0.05	0.407–4.013
LPC 18:1	LPC 18:1 d7	NA	0.999	1.000	<0.05	0.301–3.378
LPC 19:0	LPC 18:1 d7	4.17	0.995	0.992	<0.05	0.001–0.007
LPE 18:0	LPC 18:1 d7	1.78	0.993	0.998	<0.05	0.032–0.218
LPI 16:0	LPC 18:1 d7	NA	0.997	0.995	<0.05	0.000–1.304
LPI 20:4	LPC 18:1 d7	0.09	0.997	0.991	<0.05	0.009–0.093
Oleamide	LPC 18:1 d7	37.12	0.994	0.990	<0.05	0.141–0.398
Oleic acid	Palmitic acid d13	7.75	0.994	0.992	<0.05	1.289–4.806
Palmitoleic acid	Palmitic acid d13	17.03	0.997	0.997	<0.05	0.067–0.426
SPA-1P	Sphingosine d7	8.99	0.989	0.987	<0.05	0.000–0.467
Sphinganine-C17	Sphingosine d7	1.35	0.999	0.999	<0.05	0.015–1.337
Sphingosine	Sphingosine d7	0.08	0.999	0.999	<0.05	0.000–1.319
S1P	Sphingosine d7	3.41	0.991	0.986	<0.05	0.002–0.044

LPC: lysophosphocholine; LPE: lysophosphoethanolamine; LPI: lysophosphatidylinositol; SP1: sphingosine-1-phosphate; SPA-1P: sphinganine-1-phosphate. ^&^ Ratio of blank signal and metabolite standard at 100% level expressed in %.

**Table 3:** Precision and recovery parameters of HILIC and reversed-phase method.

Metabolite	Precision of the Method			Recovery of the Method		
	Intra-Assay (*n* = 6)		Inter-Assay (*n* = 12)	% (*n* = 12)	±	RSD (%)
	RSD (%) Day1	RSD (%) Day2	RSD (%)			
**HILIC Method**						
Adenosine	2.66	0.93	3.62	88.33	±	3.20
Arginine	4.52	6.99	17.17	81.42	±	8.46
Betaine	1.14	1.54	6.89	118.02	±	8.14
Carnitine	0.37	0.39	0.40	95.47	±	0.38
Cortisol	1.88	1.92	1.95	105.11	±	2.05
Creatine	4.03	1.16	8.94	99.37	±	8.89
Creatinine	4.03	1.16	8.94	99.37	±	8.89
Hexanoylcarnitine	3.33	1.34	3.08	93.80	±	2.88
Hippuric acid	3.52	2.78	3.78	100.83	±	3.81
Hypoxanthine	2.24	1.13	5.23	117.57	±	6.15
Leucine/isoleucine	1.48	1.20	1.28	94.75	±	1.22
Phenylalanine Proline	3.60	2.56	4.45	93.69	±	4.17
	4.06	2.73	4.87	99.17	±	4.83
Propionylcarnitine	0.87	0.72	1.15	96.86	±	1.11
Urea	1.94	1.05	3.83	101.77	±	3.89
**Reversed-phase method**						
Arachidonic acid	2.44	1.25	2.05	104.15	±	2.13
Bilirubin	9.69	7.39	22.29	87.92	±	21.60
Lactic acid	0.84	1.43	9.10	81.67	±	7.43
Lauric acid	5.64	4.94	5.28	97.10	±	5.12
LPC 14:0	3.05	1.94	2.44	102.63	±	2.50
LPC 16:0	1.23	0.47	1.25	102.32	±	1.28
LPC 17:0	2.38	3.01	2.61	103.79	±	2.71
LPC 17:1	4.28	3.07	4.16	102.26	±	4.25
LPC 18:0	1.46	1.78	1.57	99.76	±	1.56
LPC 18:1	1.86	1.57	1.81	100.22	±	1.81
LPC 19:0	7.81	13.67	10.64	89.15	±	9.48
LPE 18:0	2.75	3.13	3.14	100.07	±	3.14
LPI 16:0	5.74	3.09	27.64	116.24	±	9.75
LPI 20:4	46.84	65.94	56.42	50.33	±	28.19
Oleamide	4.32	2.18	6.83	33.98	±	2.32
Oleic acid	2.25	1.94	2.02	101.38	±	2.04
Palmitoleic acid	2.64	1.68	2.83	96.07	±	2.72
SPA-1P	9.60	13.12	19.19	132.39	±	4.81
Sphinganine-C17	0.89	0.96	1.66	101.79	±	1.69
Sphingosine	0.65	0.90	0.79	100.43	±	0.79
S1P	7.08	8.33	14.31	79.31	±	11.35

LPC: lysophosphocholine; LPE: lysophosphoethanolamine; LPI: lysophosphatidylinositol; SP1: sphingosine-1-phosphate; SPA-1P: sphinganine-1-phosphate.

**Table 4:** Experimental and HMDB concentrations of the analyzed metabolites.

Metabolite	HMDB Data Base (µM)	Quantified Concentrations (µM)	Included in the Range
**HILIC Method**			
Adenosine	0.01–1.71	0.04	✓
Arginine	10.00–140.00	8.30	OR
Betaine	20.00–144.00	83.07	✓
Carnitine	20.00–60.00	85.67	OR
Cortisol	0.01–0.70	204.02	OR
Creatine	32.22–80.00	73.42	✓
Creatinine	8.00–150.00	136.95	✓
Hexanoylcarnitine	0.06–0.13	0.07	✓
Hippuric acid	1.00–30.00	4.07	✓
Hypoxanthine	0.10–12.00	12.62	OR
Leucine/isoleucine	20.00–250.00	152.74	✓
Phenylalanine	16.00–166.00	79.25	✓
Proline	100.00–300.00	458.32	OR
Propionylcarnitine	0.10–0.50	0.75	OR
Urea	50.00–9000.00	113.32	✓
**Reversed-phase method**			
Arachidonic acid	2.00–600.00	5.35	✓
Bilirubin	3.00–20.00	0.01	OR
Lactic acid	740.00–2400.00	146.91	OR
Lauric acid	1.00–12.00	3.02	✓
LPC 14:0	2.00–5.00	2.59	✓
LPC 16:0	40.00–140.00	173.24	OR
LPC 17:0	0.70–3.00	2.65	✓
LPC 17:1	NA	0.30	NA
LPC 18:0	NA	51.10	NA
LPC 18:1	10.00–40.00	34.84	OR
LPC 19:0	NA	0.05	NA
LPE 18:0	NA	3.14	NA
LPI 16:0	NA	0.00	NA
LPI 20:4	NA	LA	NA
Oleamide	1500.00–3000.00	LA	NA
Oleic acid	11.00–500.00	208.24	✓
Palmitoleic acid	11.00–300.00	12.88	✓
SPA-1P	0.01–0.10	LA	NA
Sphinganine-C17	NA	LA	NA
Sphingosine	0.05	0.01	OR
S1P	0.04–0.40	0.92	OR

HMDB: human metabolome database; LA: low abundance (<10^2^); LPC: lysophosphocholine; LPE: lysophosphoethanolamine; LPI: lysophosphatidylinositol; NA: not applicable; OR: out of range; SP1: sphingosine-1-phosphate; SPA-1P: sphinganine-1-phosphate.

**Table 5:** Clinical information of patients included in the study.

	Model 1			Model 2		
	*p*-Value	ICS	UC	*p*-Value	IT	BIO
***N***	NA	6	4	NA	23	12
**Gender (%F/%M)**	NA	100.00/0.00	100.00/0.00	0.236	65.22/34.78	83.33/16.67
**Age (years)**	0.073	31.17 ± 12.09	52.25 ± 9.95	<0.001	36.57 ± 9.82	46.58 ± 7.87
**BMI**	0.944	28.18 ± 5.86	27.87 ± 7.59	0.464	26.28 ± 4.83	27.58 ± 3.56
**Smoker (%)**	0.335	16.67	0.00	0.131	0.00	8.33
**Non-smoker (%)**	NA	83.33	75.00	NA	100.00	83.33
**Ex-smoker (%)**	NA	0.00	25.00	NA	0.00	8.33
**Onset age (years)**	0.790	17.00 ± 13.36	14.77 ± 11.00	0.419	15.39 ± 11.32	12.27 ± 3.56
**Total IgE (U)**	0.800	601.50 ± 945.92	226.25 ± 176.13	0.689	464.83 ± 500.87	565.73 ± 728.78
**AC (%)**	0.400	0.00	25.00	0.002	0.00	41.67
**AH (%)**	0.133	100.00	50.00	0.003	91.30	41.67
**BD (%)**	NA	0.00	0.00	NA	0.00	0.00
**Inhaled CS/LABA (%)**	NA	100.00	100.00	<0.001	26.09	100.00
**Inhaled CS (%)**	0.133	0.00	0.00	0.009	4.35	0.00
**Topic CS (%)**	0.119	100.00	50.00	<0.001	100.00	66.67
**S (%)**	0.024	16.67	75.00	0.104	13.04	75.00
**SABA (%)**	0.400	16.67	100.00	NA	78.26	100.00
**T (%)**	NA	0.00	25.00	0.657	0.00	0.00

BMI: Body mass index; U: ISAC units; AC: anticholinergic; AH: antihistaminic; BD: bronchodilator; CS: corticosteroid; LABA: long-acting beta-adrenoceptor agonist; NA: not applicable; S: singulair (antileukotriene); SABA: short-acting beta-adrenoceptor agonist; T: theophylline; ICS: patients controlled with inhaled or topic corticosteroids without the need for systemic corticosteroids; UC: uncontrolled patients, IT: patients controlled with immunotherapy, BIO: patients controlled with omalizumab.

## Data Availability

The datasets generated during the current study are available from the corresponding author upon reasonable request as there is no online web to upload MRM data, including the raw and the curated data.

## Supplementary Materials

The following supporting information can be downloaded at: https://www.mdpi.com/article/10.3390/metabo12070592/s1, Figure S1: Chromatographic profile for HILIC method; Figure S2: Chromatographic profile for reversed-phase method; Table S1: Selected metabolites from non-targeted metabolomics studies; Table S2: Complete clinical information of patients recruited in the study; Table S3: Pairwise comparisons showing the significance of metabolites among study groups.
