# Supplementary material for: Development of a Novel Targeted Metabolomic LC-QqQ-MS Method in Allergic Inflammation
Source: Metabolites. 2022 Jun 25;12(7):592. doi: 10.3390/metabo12070592 (PMC9319984; doi:10.3390/metabo12070592)
Supplement: Supplementary file 1 [file metabolites-12-00592-s001.zip › metabolites-1776096-supplementary.pdf]

metabolites

Article

Development of a Novel Targeted Metabolomic LC-QqQ-MS Method in Allergic Inflammation

Supplementary information content:

Figure S1: Chromatographic profile for HILIC method.

Figure S2: Chromatographic profile for reversed-phase method.

Table S1: Selected metabolites from non-targeted metabolomics studies.

Table S2: Complete clinical information of patients recruited in the study.

Table S3: Pairwise comparisons showing the significance of metabolites among study groups.

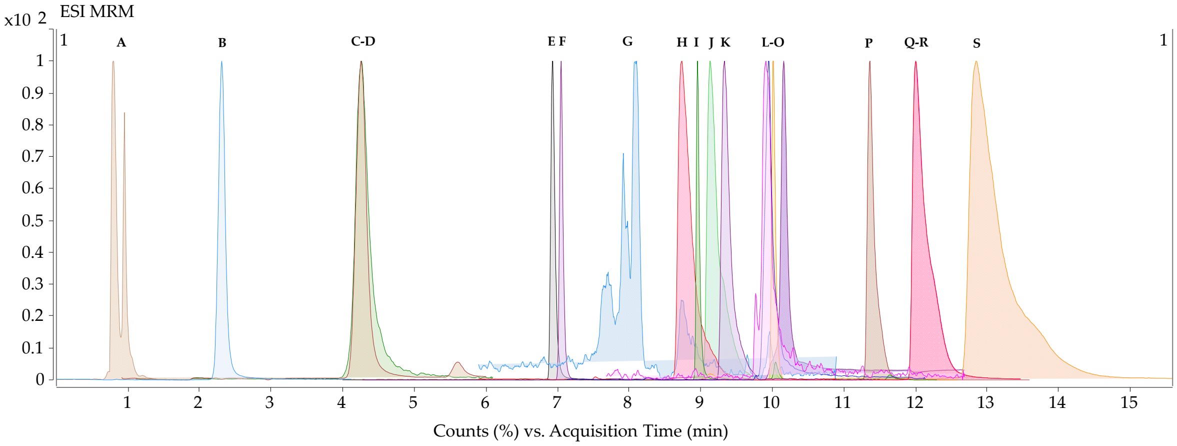

Figure S1. Chromatographic profile for HILIC method. A: Cortisol; B: Urea; C: Hypoxanthine; D: Adenosine; E: Creatinine; F: Hippuric acid; G: Phenylalanine; H: Phenylalanine d5; I: Leucine/Isoleucine; J: Isoleucine d7; K: Proline; L: Valine d8; M: Creatine; N: Betaine; O: Hexanoylarnitine; P: Propionylcarnitine; Q: Carnitine d3; R: Carnitine; S: Arginine.

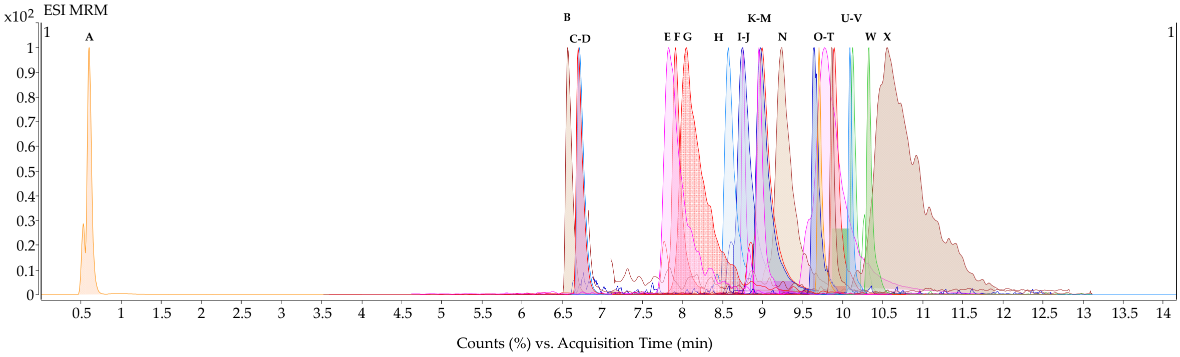

Figure S2. Chromatographic profile for reversed-phase method. A: Lactic acid; B: Sphinganine C17; C: Sphingosine; D: Sphingosine d7; E: Sphingosine-1-P; F: Sphinganine-1-P; G: LPC 14:0; H: LPC 17:1; I: LPC 16:0; J: Lauric acid; K: LPC 18:1; L: LPE 18:0; M: LPC 17:0; N: LPC 18:1 d7; O: LPC 18:0; P: LPI 20:4; Q: Palmitoleic acid; R: LPI 16:0; S: Oleamide; T: Arachidonic acid; U: Bilirubin; V: Palmitic acid d31; W: Oleic acid; X: LPC 19:0.

Table S1. Selected metabolites from non-targeted metabolomics studies.

Metabolite	Biochemical subclass	Model		
		Severe respiratory allergy	Anaphylaxis	Uncontrolled severe allergic asthma
**HILIC method**				
Adenosine	Purine nucleoside	✓		
Arginine	Amino acid		✓	✓
Betaine	Amino acid		✓	
Carnitine	Quaternary ammonium salt	✓		
Cortisol	Hydroxysteroid		✓	
Creatine	Amino acid		✓	
Creatinine	Amino acid		✓	
Hexanoylcarnitine	Fatty acid ester	✓		
Hippuric acid	Benzoic acid		✓	✓
Hypoxanthine	Purine	✓		
Leucine/Isoleucine	Amino acid	✓	✓	✓
Phenylalanine	Amino acid		✓	
Proline	Amino acid		✓	
Propionylcarnitine	Fatty acid ester	✓		
Urea	Urea	✓		
**Reversed-phase method**				
Arachidonic acid	Fatty acid			✓
Bilirubin	Bilirubin			✓
Lactic acid	Alpha hydroxy acid	✓	✓	

Lauric acid	Fatty acid	✓		
LPC 14-0	Glycerophosphocholine			
LPC 16-0	Glycerophosphocholine	✓		✓
LPC 17-0	Glycerophosphocholine			✓
LPC 17-1	Glycerophosphocholine			✓
LPC 18-0	Glycerophosphocholine	✓	✓	✓
LPC 18-1	Glycerophosphocholine	✓		✓
LPC 19-0	Glycerophosphocholine	✓		
LPE 18	Glycerophosphoethanolamine	✓		✓
LPI 16-0	Glycerophosphoinositol			✓
LPI 20-4	Glycerophosphoinositol			✓
Oleamide	Fatty amide		✓	
Oleic acid	Fatty acid	✓		
Palmitoleic acid	Fatty acid			✓
Sphinganine 1P	Amine	✓		
Sphinganine C17	Amine	✓		
Sphingosine	Amine	✓		
Sphingosine 1P	Amine	✓		✓

LPC: lysophosphocholine; LPE: lysophosphoethanolamine; LPI: lysophosphatidylinositol; SP1: sphingosine-1-phosphate; SPA-1P: sphinganine-1-phosphate.

Table S2. Complete clinical information of patients recruited in the study.

Patient	Group	Gender	Age (years)	BMI	Smoking status	Onset Age (years)	Treatment	Total IgE (U)
1	ICS	F	30	30.00	no	5	Topic CS + AH + Inhaled CS/LABA	160
2#	ICS	F	31	20.43	no	14	Topic CS +AH + BD	346
3	ICS	F	22	25.91	no	20	Topic CS + AH + Inhaled CS/LABA	76
4	ICS	F	41	34.29	yes	9	Topic CS + AH + Inhaled CS/LABA	89
5#	ICS	F	41	34.88	no	21	Topic CS + AH + Inhaled CS/LABA	611
6#	ICS	F	40	26.034	yes	11	Topic CS + AH + Inhaled CS/LABA	365
7	ICS	F	41	34.58	no	22	Topic CS + AH + Inhaled CS/LABA	237
8	ICS	F	32	24.62	no	6	Topic CS + AH + S + Inhaled CS/LABA	2500
9	ICS	F	57	19.67	no	40	Topic CS + AH + Inhaled CS/LABA + SABA	547
10#	ICS	F	43	26.13	ex-smoker	6	Topic CS + AH + Inhaled CS/LABA + SABA	206
11#	UC	F	36	26.84	no	4	S + Inhaled CS/LABA + SABA + AH + Topic CS	2000
12#	UC	F	43	28.62	no	4	Inhaled CS/LABA + AC + S + SABA	647
13#	UC	F	24	23.94	no	2	Inhaled CS/LABA + SACA + Topic CS + AH	1585
14	UC	F	48	29.48	ex-smoker	5	Inhaled CS/LABA + SABA + Topic CS + AH	455
15	UC	F	64	27.63	no	15	Inhaled CS/LABA + SABA + S + T	160
16	UC	F	56	30.08	no	18	Inhaled CS/LABA + SABA + Topic CS + AH + S	45
17	UC	F	41	36.39	no	9	S + Inhaled CS/LABA + AC + SABA	245
18#	IT	F	39	21.60	no	25	Topic CS + AH + Inhaled CS/LABA	208
19#	IT	F	39	23.72	no	20	Topic CS + AH + Inhaled CS/LABA	231
20#	IT	F	31	24.97	no	12	Topic CS + AH + Inhaled CS/LABA	182

21#	IT	F	35	25.78	no	29	Topic CS + AH SABA	94
22#	IT	F	28	21.97	no	3	Topic CS + AH + SABA	191
23#	IT	F	43	28.93	no	28	Topic CS + SABA	331
24	IT	F	31	37.95	no	16	Topic CS + AH + SABA	285
25	IT	M	29	28.06	no	14	Topic CS + SABA	741
26	IT	F	47	29.27	no	32	Topic CS + AH + SABA	279
27#	IT	F	44	24.54	no	15	Topic CS + AH + SABA	981
28#	IT	M	56	32.56	no	47	Topic CS + AH + SABA	96
29#	IT	F	38	26.81	no	15	Topic CS + AH + SABA	170
30	IT	F	34	35.06	no	28	Topic CS + AH + SABA	41
31	IT	F	43	30.33	no	14	Topic CS + AH + Inhaled CS/LABA	303
32	IT	M	44	26.58	no	34	Topic CS + AH + SABA	256
33	IT	F	42	23.73	no	30	Topic CS + AH + S + SABA	49
34	IT	F	42	20.83	no	34	Topic CS + AH + SABA	581
35	IT	F	24	21.99	no	3	Topic CS + AH + SABA	1217
36	IT	M	22	18.67	no	10	Topic CS + AH + SABA	778
37	IT	F	45	29.73	no	11	Topic CS + SABA + Inhaled CS	135
38#	IT	M	22	24.19	no	7	Topic CS + AH + Inhaled CS/LABA	1360
39	IT	M	42	28.53	no	5	Topic CS + AH + Inhaled CS/LABA	687
40	IT	F	21	22.78	no	4	Topic CS + AH + SABA	182
41	IT	F	39	22.04	no	12	Topic CS + AH + SABA	149
42#	IT	F	59	24.44	no	35	Topic CS + S + Inhaled CS/LABA	165
43	IT	F	36	24.69	no	2	Topic CS + AH + SABA	2000

44	IT	F	40	22.14	no	27	Topic CS + AH + Inhaled CS/LABA	168
45	IT	F	23	21.91	no	5	Topic CS + AH + SABA	424
46	IT	M	44	29.59	no	25	Topic CS + AH + SABA	244
47	IT	F	60	26.22	no	26	Topic CS + AH + S + Inhaled CS/LABA + SABA	219
48#	IT	F	34	32.02	no	14	Topic CS + AH + SABA	192
49#	IT	F	40	23.62	no	17	Topic CS + AH + SABA	184
50#	IT	F	42	29.73	no	12	Topic CS + AH + SABA	292
51#	IT	F	49	22.07	no	13	Topic CS + AH + Inhaled CS/LABA + S + SABA	52
52	IT	M	22	20.52	no	4	Topic CS + AH + S + SABA	1502
53	IT	M	41	24.82	no	5	Topic CS + AH + SABA	220
54#	IT	F	31	20.08	no	8	Topic CS + AH + Inhaled CS/LABA + SABA	178
55#	IT	F	22	29.39	no	4	Topic CS + AH + Inhaled CS/LABA + SABA	61
56#	IT	F	41	24.28	no	18	Topic CS + AH + SABA	660
57#	IT	F	24	21.16	no	15	INHALED CS/LABA + Topic CS + AH	96
58	IT	M	33	27.78	no	7	Topic CS + AH + Inhaled CS/LABA	77
59#	IT	F	38	27.83	no	5	AH + SABA	532
60	IT	F	37	31.24	no	6	Topic CS + AH + Inhaled CS/LABA	154
61#	IT	F	44	29.34	ex-smoker	11	Topic CS + AH + SABA	487
62	BIO	M	43	28.73	ex-smoker	5	Inhaled CS/LABA + SABA + S	39
63#	BIO	F	40	20.24	no	6	Inhaled CS/LABA + SABA + S	358
64	BIO	F	61	26.89	no	11	Inhaled CS/LABA + SABA	93
65	BIO	F	49	33.98	no	14	Inhaled CS/LABA + SABA + S	170
66	BIO	F	38	25.56	no	&	Inhaled CS/LABA + SABA + S + AC	&

67	BIO	F	55	31.53	no	20	Inhaled CS/LABA + SABA + S + Topic CS + AH	306
68	BIO	F	47	30.46	no	24	Inhaled CS/LABA + SABA + S + Topic CS	2000
69#	BIO	F	40	33.86	no	23	Inhaled CS/LABA + S + SABA + Topic CS + AC	281
70	BIO	F	37	26.72	yes	6	Inhaled CS/LABA + S + SABA + AC + Topic CS + AH	606
71#	BIO	F	22	23.88	no	14	Inhaled CS/LABA + S + AC + Topic CS + AH + SABA	321
72	BIO	F	39	23.26	no	11	Inhaled CS/LABA + S + SABA + Topic CS + AC	43
73	BIO	F	44	21.31	no	12	Inhaled CS/LABA + S + SABA + Topic CS + AC	2000
74	BIO	F	54	24.91	no	21	Inhaled CS/LABA + SABA + Topic CS + AH	210
75	BIO	F	39	28.84	no	5	Inhaled CS/LABA + SABA + Topic CS + AH	386
76	BIO	M	53	28.73	no	6	Inhaled CS/LABA + SABA + AC + S + Topic CS + AH	370

BMI: Body mass index; U: ISAC units; AC: anticholinergic; AH: antihistaminic; BD: bronchodilator; CS: corticosteroid; LABA: long-acting beta-adrenoceptor agonist; S: singulair (antileukotriene); SABA: short-acting beta-adrenoceptor agonist; T: theophylline; ICS: Patients controlled with inhaled or topic corticosteroids without the need for systemic corticosteroids; UC: Uncontrolled patients; IT: Patients controlled with immunotherapy; BIO: Patients controlled with Omalizumab. #Patients did not pass TUS quality filter. &Data not available.

Supplementary Table S3. Pairwise comparisons showing the significance of metabolites among study groups.

Metabolite	Model 1<br>UC vs ICS	Model 2<br>BIO vs IT
	p-value	*p-value*
**HILIC method**		
Adenosine	0.114	0.231
Arginine	0.352	0.476
Betaine	0.762	0.205
Carnitine	0.610	0.741
Cortisol	0.914	0.566
Creatine	0.914	0.689
Creatinine	0.914	0.848
Hexanoylcarnitine	0.352	0.322
Hippuric acid	0.067	0.231
Hypoxanthine	0.257	0.664
Leucine/isoleucine	0.171	**0.049**
Phenylalanine	0.171	0.794
Proline	0.762	0.794
Propionylcarnitine	0.257	0.794
Urea	0.914	0.566
**Reversed-phase method**		
Arachidonic acid	0.257	0.543
Bilirubin	0.352	0.395

Lactic acid	1.000	0.741
Lauric acid	0.610	NA
LPC 14:0	**0.010**	**0.001**
LPC 16:0	0.067	0.217
LPC 17:0	**0.038**	0.614
LPC 17:1	**0.019**	**0.014**
LPC 18:0	0.067	**0.009**
LPC 18:1	0.476	0.768
LPC 19:0	NA	NA
LPE 18:0	**0.038**	0.193
LPI 16:0	NA	0.217
LPI 20:4	NA	NA
Oleamide	NA	NA
Oleic acid	0.762	0.414
Palmitoleic acid	0.610	0.244
SPA-1P	NA	NA
Sphinganine-C17	0.114	NA
Sphingosine	0.476	NA
S1P	0.257	0.566

LPC: lysophosphocholine; LPE: lysophosphoethanolamine; LPI: lysophosphatidylinositol; NA: not applicable; SP1: sphingosine-1-phosphate; SPA-1P: sphinganine-1-phosphate. Significant differences are marked **in bold**.
